# Targeting Pannexin1 Improves Seizure Outcome

**DOI:** 10.1371/journal.pone.0025178

**Published:** 2011-09-16

**Authors:** Marcelo F. Santiago, Jana Veliskova, Naman K. Patel, Sarah E. Lutz, Dorothee Caille, Anne Charollais, Paolo Meda, Eliana Scemes

**Affiliations:** 1 The Dominick P. Purpura Department of Neuroscience, Albert Einstein College of Medicine, New York, New York, United States of America; 2 Instituto de Biofisica Carlos Chagas Filho, Universidade Federal do Rio de Janeiro, Rio de Janeiro, Rio de Janeiro, Brazil; 3 The Saul R. Korey Department of Neurology, Albert Einstein College of Medicine, New York, New York, United States of America; 4 Department of Cell Physiology and Metabolism, University of Geneva, Geneva, Switzerland; Dalhousie University, Canada

## Abstract

Imbalance of the excitatory neurotransmitter glutamate and of the inhibitory neurotransmitter GABA is one of several causes of seizures. ATP has also been implicated in epilepsy. However, little is known about the mechanisms involved in the release of ATP from cells and the consequences of the altered ATP signaling during seizures. Pannexin1 (Panx1) is found in astrocytes and in neurons at high levels in the embryonic and young postnatal brain, declining in adulthood. Panx1 forms large-conductance voltage sensitive plasma membrane channels permeable to ATP that are also activated by elevated extracellular K^+^ and following P2 receptor stimulation. Based on these properties, we hypothesized that Panx1 channels may contribute to seizures by increasing the levels of extracellular ATP. Using pharmacological tools and two transgenic mice deficient for Panx1 we show here that interference with Panx1 ameliorates the outcome and shortens the duration of kainic acid-induced status epilepticus. These data thus indicate that the activation of Panx1 in juvenile mouse hippocampi contributes to neuronal hyperactivity in seizures.

## Introduction

Among the various types of paracrine signals, purinergic ATP-mediated signaling is emerging as one of the most prominent form involved in neural cell interactions (reviewed in [1]). This is because all neural cell types are able to release and respond to ATP and/or its metabolites. Purinergic signaling is not only involved in physiological glia-glia and glia-neuronal communication, but also plays critical roles in events related to epileptogenesis. Generalized seizures were reported after microinjection of ATP into the pre-piriform cortex and augmented levels of ATP were measured in hippocampi of mice with audiogenic seizures ([2]; but see [3]; reviewed in [4]).

Recently, Pannexin1 (Panx1) in pyramidal neurons was found to contribute to NMDA-mediated epileptiform activity by increasing spike amplitude and decreasing burst intervals [5]. Pannexins (Panx 1, 2, and 3) are a group of proteins that share sequence homologies with the invertebrate gap junction proteins, the innexins. Differently, however, from their homologues, pannexins do not form gap junction intercellular channels, but do form plasma membrane channels, at least in the case of Panx1. Panx1 transcripts have been shown to be expressed in the CNS, at high levels in early stages of murine brain development, decreasing in adulthood [6].

When exogenously expressed, Panx1 forms large conductance (500pS) channels that are permeable to ATP [7]. These channels have been reported to be activated by voltage, by mechanical stretch, by elevated extracellular K^+^, and following P2 receptor activation [8], [9], [10]. Moreover, Panx1 is associated with the P2X_7_ receptor [11], [12], and, at least in astrocytes, this association is essential for the ATP-induced ATP release mechanism involved in the transmission of calcium signals among these cells [13], [14], [15].

Intense neuronal activity, such as that occurring during epileptiform convulsions can elevate extracellular K^+^ to levels that promote opening of Panx1 channels. Here we tested the hypothesis that Panx1 channels contribute to seizures by releasing the excitatory nucleotide ATP, using a Panx1 channel blocker and two different Panx1-null mice (Panx1^−/−^ and Panx1 KOfirst). Our results indicate that Panx1 channels are opened by elevated K^+^ concentration and following kainic acid-induced status epilepticus and that blockade or deletion of Panx1 reduces the amount of ATP that is released and improves the behavioral manifestation of seizures.

## Materials and Methods

### Ethics Statement

Mice were housed and maintained under specific pathogen-free conditions in the Animal Resource Facilities of the University of Geneva and the Albert Einstein College of Medicine and all experiments were pre-approved by the Animal Care and Use Committee (IACUC approval numbers AUP # 2010-0414 and 1034/3552/1).

### Animals

Wild-type and Panx1-null mice (postnatal day 13–14), all on the C57Bl/6 background, were used (Fig. S1). All procedures were according to standard animal welfare protocol, as authorized by the local veterinary authorities. We used two different Panx1 transgenic mice, the Panx1^−/−^ mice generated by Dr. Hannah Monyer [16] and the heterozygote Panx1-knockout (KO)first (Panx1^tm1a(KOMP)Wtsi^) mice generated and purchased from KOMP at UCDavis, an NIH initiative. Panx^+/−^ mice were maintained at the animal facility at the University of Geneva. Panx1 KOfirst heterozygotes were bred to homozygosity and maintained in the animal facility at Albert Einstein College of Medicine.

### Genotyping

Panx1^−/−^ mice were genotyped by PCR of tail DNA (0.5 µl) using 3 primers (**a**: 5′GGAAAGTCAACAGAGGTACCC3′; **b**: 5′CTTGGCCACGGAGTATGTGTT3′; **c**: 5′GTCCCTCTCACCACTTTTCTTACC3′). The wild type Panx1 allele was targeted by primers **a** and **b**, and was identified by a 330 bp amplicon, whereas the mutated allele was targeted by primers **a** and **c**, and was identified by a 630 bp amplicon (Fig. S1). Real-time quantitative polymerase chain reaction was used to amplify RNA extracted from the hippocampi of 3 mice per genotype using RNeasy® Micro Kit (Qiagen AG, Basel, Switzerland). RNA samples were then treated with DNase I. One µg total RNA was reverse transcribed using random hexamers Superscript III reverse transcriptase (Invitrogen, Grand Island, NY, USA), and amplified using single stranded oligonucteotides for Panx1 (sense: TATTGCCGTGGGTCTACCTC; antisense: TGTCGCCAGGAGAAAGAACT) designed for SYBR green assays using the program Primer Express v 2.0 (Applied Biosystems, Foster City, CA, USA) and a SDS 7900 HT instrument (Applied Biosystems). Each reaction was performed in three replicates on 384-well plates. Raw Ct values obtained with SDS 2.2 (Applied Biosystems) were normalized to the geometric mean of 3 stable genes (eef1a1, gapdh, gusb), and used to calculate fold-changes by the GeNorm method.

Panx1 KOfirst were genotyped by tail PCR using 4 primers (**1**: 5′GAGATGGCGCAACGCAATTAAT3′; **2**: 5′CTGGCTCTCATAATTCTTGCCCTG3′; **3**: 5′ CTGTATCACACAACCACTTCAGAGAAGG3′; **4**: 5′GAGCTGACCCCTTTCCATTCAATAG3′). The wild type Panx1 allele was targeted by primers **3** and **4** and identified as a 579 bp amplicon, while the transgene was targeted by primers **1** and **2** and identified as a 381 bp amplicon (Fig. S1). Western blots were used to evaluate Panx1 protein deletion in cultured brain astrocytes of Panx1 KOfirst (Fig. S1).

### Immunohistochemistry

Coronal cryosection of p-formaldehyde fixed hippocampi were immunostained using anti-Panx1 (Aves Lab), anti-GFAP (Sigma), and anti-NeuN (Milipore) antibodies. Isotype specific, Alexa Fluo secondary antibodies were used to identify the cellular distribution of these proteins using a Zeiss LSM 510 Duo confocal microcope equipped with a 63× oil immersion objective and META spectral detection system.

### Western blot

Cell lysates of cultured astrocytes derived from WT and Panx1 KOfirst neonate mouse brains were electrophoresed in 4–20% mini-gels. After transfer of proteins to nitrocellulose membranes and one hour incubation in blocking phosphate buffered solution (PBS) containing 0.5% Tween-20 and 2% skinned milk, blotting was performed for two hours at room temperature (RT) using anti-Panx1 antibodies (1∶1000; Aves Lab) and for 1 hr when using anti-GAPDH (Fitzgerald). After washes with PBS-Tween-20, membranes were incubated with goat anti-chicken or goat anti-mouse HRP-conjugated secondary antibodies (1∶2000; Santa Cruz Technology) for one hour at RT. Visualization of bands was performed using X-ray film and a developer.

### Brain slices

Coronal sections (350 µm thick) of P13–P14 mouse brains were obtained using a tissue chopper (Ted Pella Inc.). After cutting, brain slices were immediately placed in Hepes buffered, air-bubbled ACSF (145 mM NaCl, 2.5 mM KCl, 3.1 mM CaCl_2_, 1.3 mM MgCl_2_, 10 mM glucose, 10 mM Hepes, pH 7.4), and maintained at 30°C. After 30–40 min, ACSF was exchanged for the appropriate ACSF necessary for each experimental condition (see below).

### Dye uptake

Panx1 activation was evaluated in acute hippocampal slices using a modified method of dye uptake, previously described [11], [13], [15]. Briefly, brain slices were incubated for 1 hr, at 30°C, in Hepes-buffered air-bubbled ACSF containing the dye (5 µM), in the absence and presence of mefloquine (MFQ: 100 nM; BioBlocks QU-024). After that, YoPro solution was removed and slices washed 3 times, 10 min each with 5 ml ACSF. After the last wash, brain slices were fixed overnight, at 4°C, in 4% p-formaldehyde, and then transferred to ice-cold ACSF containing 30% sucrose for few hours and hippocampal slices isolated from the surrounding brain tissue. YoPro1 uptake in hippocampi following exposure to ACSF containing 2.5 mM and 10 mM K^+^ concentrations (osmolarity adjusted with equimolar concentration of NaCl) and after kainic acid induced status epilepticus was measured from regions of interest placed on the pyramidal cell layer (CA1–CA3) and stratum radiatum of p-formaldehyde fixed tissues using an Nikon inverted microcope equipped with 4× objective, 488/512 nm filter sets and Metafluor software.

### ATP release

The amount of ATP released into the ACSF by acute brain slices was measured as previously described [15], [17], [18], using the luciferin/luciferase assay (Molecular Probes and Promega) and a Turner luminometer. Fifty µl of the 5 ml ACSF bathing the brain slices were collected every 15 min during one hour and stored at −20°C until use. After that time, brain slices were sonicated in lysis buffer (150 mM NaCl; 10 mM Tris-base; 1% TritonX-100; protease inhibitor cocktail; pH 7.4) and total protein measured using the BCA reagents (Thermo Scientifc). The concentrations of ATP released from brain slices were obtained from standard curves and normalized to the total amount of protein.

### Kainic acid-induced seizures

Mice were injected intraperitoneally (i.p.) with kainic acid (3.2–3.6 mg/kg body weight; Sigma) and maintained in a temperature controlled environment for observation of seizures. Seizure monitoring was done for up to 2 hours after the onset of status epilepticus (SE). In some experiments, mice were injected i.p. with the Panx1 channel blocker MFQ (0.4 µg/kg body weight) 2 hr prior to kainic acid injection. After experimentation, mice were euthanized by decapitation and coronal brain sections collected for further functional testing.

To determine the effective kainic acid concentration necessary to induce sustained status epilepticus for at least two hours in juvenile (P13–P14) mice, we injected a range (3.0–4.0 mg/kg) of kainic acid concentration. The dose that produced 2 hrs of sustainable SE and the lowest mortality was then chosen. We used 3.6 mg/kg body weight to inject WT and Panx1 KOfirst mice. Behavioral analysis was performed simultaneously by two observers when evaluating KA-induced SE in WT and Panx1 KOfirst mice and the effects of Panx1 blocker MFQ on KA-induced SE in WT mice. Control experiments were always performed in parallel (saline-injected *vs* KA-injected WT mice, and MFQ- and KA-injected *vs* KA-injected mice). Blind experiments were performed when using Panx1^−/−^ transgenic mice. In this case, litters derived from Panx1^+/+^×Panx1^+/−^ and from Panx1^+/−^×Panx1^−/−^ breeding were tested prior to animal genotyping. Due to the remodeling of the animal facility at the University of Geneva that occurred during the experimentation period and influenced mouse breeding and behavior, the dose of 3.6 mg/kg kainic acid used initially had to be re-adjusted to 3.2 mg/kg to avoid a high incidence of animal mortality. Data obtained from the initial studies using 3.6 mg/kg KA-injected Panx1^+/+^ and Panx1^−/−^ mice did not differ statistically from those obtained from 3.2 mg/kg KA-injected Panx1^+/+^ and Panx1^−/−^ mice and therefore were grouped for analyses. We used at Albert Einstein a total of 33 WT and 6 Panx1 KOfirst mice. Of the 51 P13-P14 Panx1 transgenic mice tested in Geneva, 12 were Panx1^+/+^, 26 were Panx1^+/−^ and 13 were Panx1^−/−^. Data obtained from animals that died (2 Panx1^+/+^, 3 Panx1^+/−^, 3 Panx1^−/−^) during the course of 3.6 mg/kg KA-induced SE were discarded from analyses.

## Results

### Hipocampal neurons and astrocytes express pannexin1

**Figure 1:**
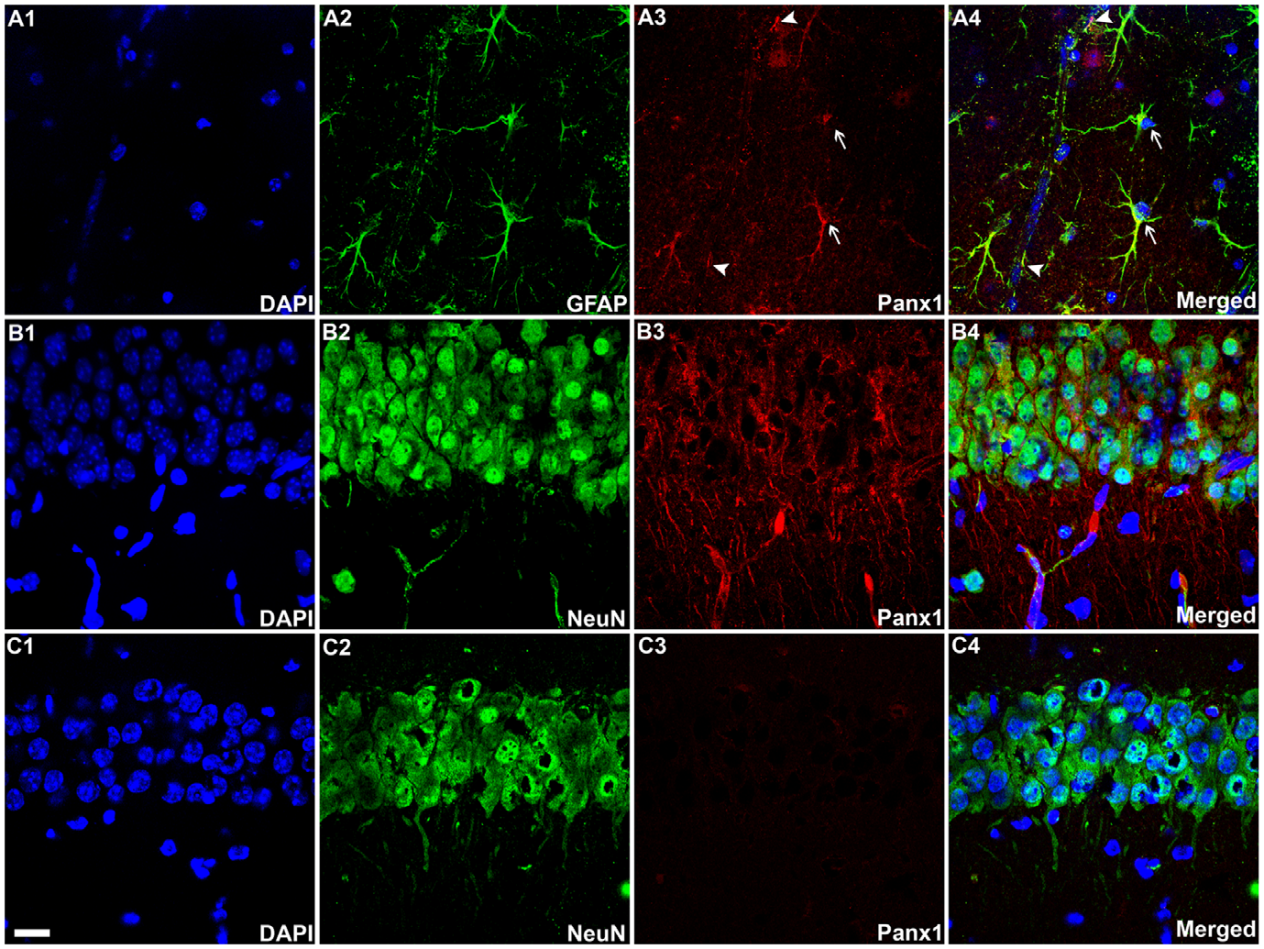
Pannexin1 is expressed in astrocytes and neurons in the hippocampus. Confocal images of the stratum radiatum (A1–A4) and pyramidal cell layer (B1–B4) of the CA1 hippocampal region of P14 WT mice showing that GFAP-positive astrocytes and NeuN-positive hippocampal neurons express Panx1; nuclei were counterstained with DAPI. Note in (C1–C4) the lack of staining in the pyramidal cell layer from Panx1^−/−^ mice. Merged images are shown in A4, B4 and C4. Arrows and arrow heads in A3–4 indicate Panx1 staining in astrocyte cell bodies and endfeet, respectively. Bar: 15 µm.

Although Panx1 transcripts have been detected in the rodent CNS [6], [19], [20], there is only one report indicating Panx1 protein expression in hippocampal and cortical neurons *in situ* [21]. Yet, Panx1 expression in astrocytes has only been observed in cell culture preparations [15], [22]. Our immunohistochemistry performed on P14 mouse hippocampi confirmed the presence of Panx1 in both neurons and astrocytes, as identified by NeuN and GFAP positive cells, respectively, and its absence in Panx1^−/−^ mice (Fig. 1). Note in WT animals the intense Panx1 staining on cell bodies of neurons in the pyramidal cell layer of the CA1 region of the hippocampus and on stratum radiatum astrocyte cell bodies as well as along the astrocyte endfeet surrounding blood vessels. Further characterization of these Panx1^−/−^ mice (tail PCR and qRT-PCR) as well as the Panx1 KOfirst (tail PCR and western blot) are displayed in Figure S1.

### High extracellular potassium opens Panx1 channels in hippocampal slices

**Figure 2:**
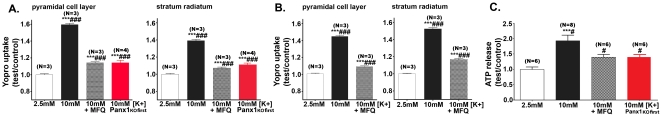
Blockade or deletion of Panx1 channels prevents membrane permeabilization of hippocampal neurons and astrocytes and attenuates ATP release from brain slices. Bar histograms showing the fractional mean ± s.e.m. values (test/control: normalized to 2.5 mM K^+^ ACSF) of YoPro1 uptake induced by 10 mM K^+^ in the pyramidal cell layer and stratum radiatum obtained in the absence and following (A) slice incubation and (B) animal injection with MFQ. A substantial reduction in dye uptake was recorded from slices of Panx1 KOfirst mice exposed to high K^+^ ACSF (red bars). (C) Bar histograms showing the fractional mean ± s.e.m. amounts of ATP present in the bathing solution of hippocampal slices exposed for 1 hr to ACSF containing either 2.5 or 10 mM K^+^. Note the increased release of ATP in 10 mM K^+^ and its reduction following the blockade (MFQ: 100 nM) and deletion (Panx1 KOfirst mice) of Panx1 channels. Data displayed in panels A and B are from 3 wild type and 4 Panx1 KOfirst mice per group. The number of animals used in part C is in parentheses. ***P<0.001 and *P<0.05 between control and test conditions; ###P<0.001, ##P<0.01, and #P<0.05 between test conditions. P values were obtained from One-way Anova analysis of variance followed by Bonferroni's multiple comparison tests.

**Figure 3:**
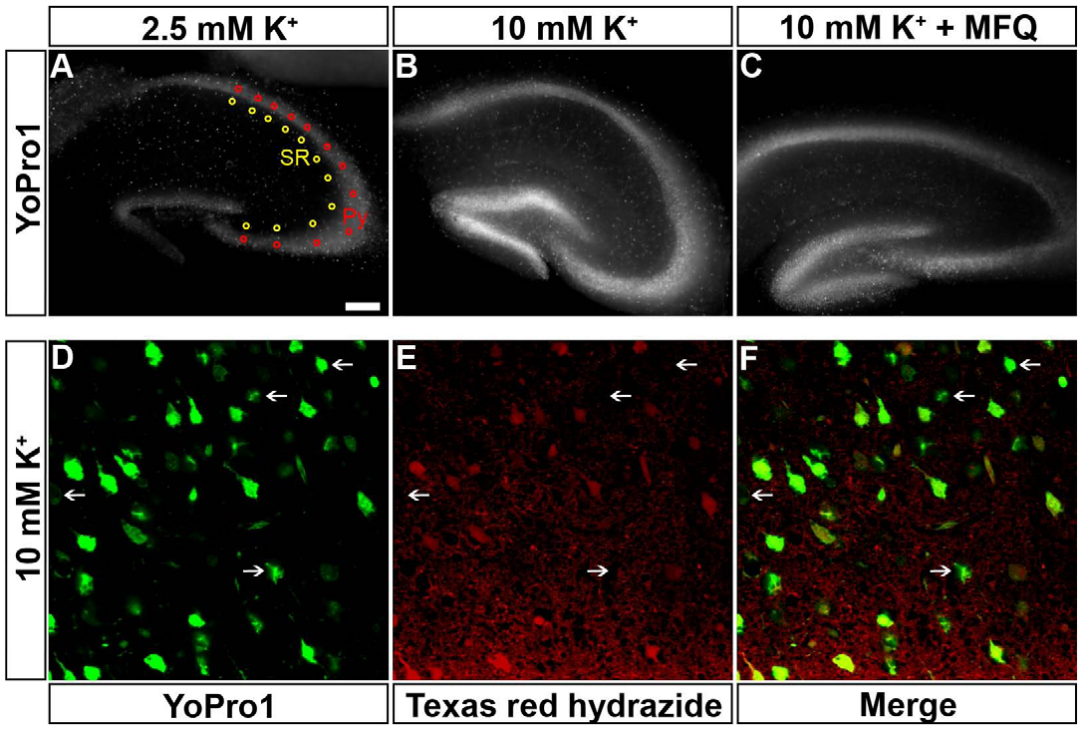
Dye uptake in hippocampal astrocytes. (A–C) Epifluorescence images of 4% p-formaldehyde fixed hippocampal slices derived from WT mice that were exposed for 1 hr to (A) 2.5 mM and to (B–C) 10 mM K^+^ ACSF containing 5 µM YoPro1 in the absence (B) and presence (C) of 100 nM MFQ. YoPro1 fluorescence intensity was measured from regions of interest placed along the neuronal cell bodies in the pyramidal cell layer (Py; red circles) and in the stratum radiatum (SR; yellow circles) of the hippocampal CA1–CA3 regions. Images were acquired using an inverted epifluorescence Eclipse Nikon microscope equipped with 4× objective controlled by Metafluor software. (D) Confocal images of cells located in the stratum radiatum of the mouse hippocampus that was exposed to 10 mM K^+^ ACSF containing YoPro1 and the fixable astrocyte marker sulforhodamine 101 (Texas Red hydrazide), showing that the great majority of YoPro1-positive cells were also stained with Texas Red. Arrows indicate a few YoPro1-positive cells in the stratum radiatum that did not incorporate Texas Red (likely interneurons). Bar: 150 µm for top panels and 25 µm for bottom panels.

We evaluated whether Panx1 channels were activated under conditions of neuronal hyperactivity by measuring the cellular influx of YoPro1 in hippocampal slices exposed for 1 hr to artificial cerebrospinal fluid (ACSF) containing 10 mM K^+^. Under these conditions, a significantly higher YoPro1 uptake in both the hippocampal pyramidal cell layer and stratum radiatum was recorded compared to that measured in normal (2.5 mM K^+^) ACSF (Fig. 2). Under elevated extracellular K^+^, the amount of dye uptake measured in pyramidal neurons was substantially higher than in stratum radiatum astrocytes (Figs. 2A–B). Evidence that YoPro1 stained cells in the stratum radiatum are astrocytes was obtained by staining the slices with Texas Red hydrazide, a fixable form of the astrocyte marker, sulforhodamine101 [23]. As shown in Figure 3, the great majority of YoPro1 positive cells in the stratum radiatum were also TxR positive cells; very few YoPro1 positive cells did not display the astrocyte marker which were likely interneurons.

Evidence that Panx1 mediated the YoPro1 dye influx was obtained by blocking Panx1 with mefloquine (MFQ) which we previously found to block these channels [24] and by using Panx1-KOfirst mice. As shown in Fig. 2A, incubation of hippocampal slices for 1 hr in ACSF (10 mM K^+^) containing YoPro1 and 100 nM MFQ greatly reduced the influx of the dye. Similar observations were made after intraperitoneal injection of MFQ (0.4 µg/kg) in P14 mice, 2 hr before brains were removed and hippocampal slices incubated for 1 hr with ACSF containing 10 mM K^+^ (Fig. 2B). As expected, compared to WT significantly lower dye uptake was recorded from hippocampal slices of Panx1 KOfirst mice exposed to elevated K^+^ ACSF (Fig. 2A). Under this condition, we also found augmented levels of ATP in the 5 ml of ACSF bathing the slices of WT mice which increased 1. 9 fold from a basal level of 0.16±0.01 to 0.31±0.01 nM ATP/µg protein when the slices were exposed to high K^+^ ACSF (Fig. 2C). Blockade or deletion of Panx1 channels attenuated the amount of ATP release induced by 10 mM K^+^. In both of these cases, high K^+^ ACSF caused a 1.4 fold increase in the amount of ATP released (MFQ: 0.22±0.01 nM ATP/µg protein, N = 6 animals; Panx1 KOfirst: 0.22±0.01 nM ATP/µg protein, N = 6 animals) (Fig. 2C). No significant difference was obtained when the amount of ATP released from MFQ-treated WT and Panx1 KOfirst slices were compared. These results support the hypothesis that elevated extracellular K^+^ as occurs during epileptiform discharges [25] activates Panx1 channels leading to increased YoPro1 uptake and ATP release.

### Pannexin1 channels are activated during status epileticus

**Figure 4:**
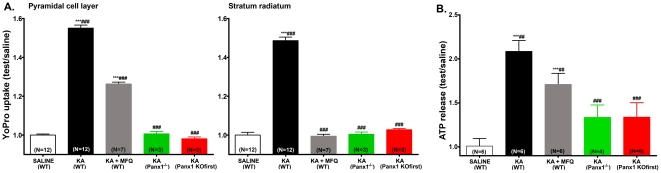
Panx1 channels contribute to membrane permeabilization during status epilepticus in mice. (A) Bar histograms showing the fractional mean ± s.e.m. values of YoPro1 uptake in pyramidal cell layer and stratum radiatum of hippocampi of WT mice that were injected with saline (white bars), KA (black bars), MFQ and KA (gray bars), from hippocampi of KA-injected Panx1^−/−^ (green bars) and Panx1 KOfirst (red bars) mice. (B) Bar histograms of the fractional mean ± s.e.m. values of ATP present in ACSF solutions bathing brain slices of saline-injected (white bar) and KA-injected (black and hashed bars) WT mice in the absence (black bar) and presence (gray bar) of 100 nM MFQ, and from brain slices of Panx1^−/−^ (green bars) and Panx1 KOfirst mice (red bars). Numbers in parentheses in parts A and B are the numbers of animals used. ***P<0.001 between control and test conditions; ###P<0.001 and ##P<0.01 between test conditions. Significance calculated using One-way Anova analysis of variance followed by Bonferroni's multiple comparison tests.

Evidence that Panx1 is activated during epileptiform activity was obtained by measuring the influx of YoPro1 in hippocampal slices from juvenile P13–14 mice in which status epilepticus (SE) was induced by i.p. injection of kainic acid (KA: 3.6 mg/kg). Compared to saline-injected mice, a significant increase in YoPro1 uptake was measured in the pyramidal cell layer and in stratum radiatum 2 hrs after KA-induced SE (Fig. 4). This effect was largely reduced by i.p. injection of MFQ (0.4 µg/kg), 2 hrs prior to KA injection (Fig. 4A gray bars). To further test whether this increase in dye uptake was mediated by Panx1 channels, we injected KA in Panx1^−/−^ and in Panx1 KOfirst mice. We found that hippocampal slices from these animals did not feature the uptake of YoPro1 observed in KA-injected wild-type (WT) mice (Fig. 4A, compare black, with either red or green bars).

Two hours after the onset of SE, extracellular ATP levels recorded from ACSF bathing the slices of KA-injected WT mice were significantly increased 2.09 fold over the values recorded from saline-injected mice (Fig. 4B). Under this condition, extracellular ATP levels increased from a basal level of 0.18±0.02 nM/µg protein recorded from slices of saline injected mice to a level of 0.36±0.02 nM/µg protein when measured from slices of KA-injected mice. After *in vitro* incubation of slices from KA-injected mice with 100 nM MFQ, the amount of ATP was reduced to 0.29±0.02 nM/µg protein, a level corresponding to 1.7 fold the basal level (Fig. 4B). The amount of ATP measured from slices of KA-injected Panx1 KOfirst mice (0.20±0.02 nM/µg protein) was 1.3 fold basal levels (Fig. 4B), which was not significantly different to that of KA-injected Panx1^−/−^ (0.21±0.03 nM/µg protein). These data show that Panx1 channels are activated during *in vivo* neuronal hyperactivity, likely contributing to the non-vesicular release of ATP which increases the extracellular levels of this excitatory transmitter.

### Pannexin1 contributes to status epilepticus: A behavioral analysis

To determine the role of Panx1 in the behavioral manifestations of SE, we evaluated whether blockade of Panx1 channel with MFQ or its deletion affected the onset, the degree, and the final behavioral outcome of seizures. For that, we used the same paradigm employed to measure dye influx in hipocampal slices, namely i.p. injection of KA, to evaluate the behavioral manifestations of seizures induced by KA. The SE scoring system used was: 0 (normal), 1 (frozen and leaning), 2 (scratching), 3 (forelimb stiff), 4 (unilateral forelimb clonus), 5 (bilateral forelimb clonus), 6 (fore- and hind-limb clonus), and 7 (tonic clonic seizures).

Besides registering the time course of seizure evolution, we also used the following parameters to evaluate the behavioral manifestations of KA-induced seizures: (a) overall seizure scores recorded during the observation period, as measured by the area under the curve (seizure score×time), (b) the time necessary to display middle range seizure score, as being the time to the onset of stage 3 (forelimb stiff), (c) the worst seizure score recorded any time during the observation period, and (d) the end point of seizure scoring.

**Figure 5:**
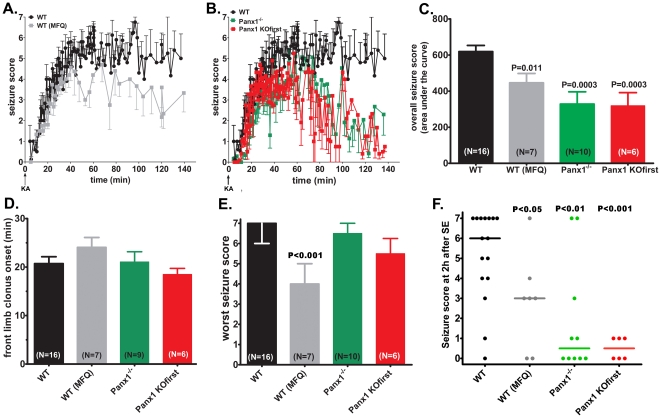
Targeting Panx1 channels ameliorates seizure outcome. (A, B) Time courses of the mean ± s.e.m. values of seizure scores measured after intraperitoneal injection of KA in (A) WT mice untreated (black symbols) and MFQ-treated (gray symbols) mice, in (B) WT (black symbols) and in two Panx1-null (Panx1^−/−^: green symbols, Panx1 KOfirst: red symbols) mice. (C) Bar histograms of the mean ± s.e.m. values obtained for the overall seizure scores (score×time: area under the curves) displayed on the left panels. Note in panel C that MFQ-treated WT mice and the two Panx1-null mouse lines displayed significant behavioral improvements (smaller areas under the curve) compared to WT mice and that the overall seizure severity of MFQ-treated WT mice were not significantly different from those of two Panx1-null mouse lines. (D–E) Bar histograms of the (D) mean ± s.e.m. values of the onset time of forelimb stiff, and of the median ± interquartile range values of (E) the worst seizure score recorded during the time course of observation. Numbers in parentheses are the number of animals used in each group. Part F shows the scatter dot plots with their respective median values of the seizure score at 2 hr after SE of KA-injected (black), MFQ- and KA -injected WT mice (gray), and KA-injected Panx1-null (Panx1^−/−^: green, Panx1 KOfirst: red) mice. Values in parts C and D are mean ± s.e.m. and the indicated P values were obtained from One-way Anova analysis of variance followed by Newman-Keuls multiple comparison test. Values in part E are medians with interquartile range, and horizontal lines in part F are the median values obtained for the scatter dot plots. Indicated P values in parts E and F were obtained from non-parametric statistical analysis Kruskal-Wallis of variance followed by Dunn's test. In part D, only 9 animals were analyzed because one of ten Panx1^−/−^ mice did not display forelimb stiff.

As shown in Figure 5A, soon after KA injection, WT mice started displaying behavioral manifestations of seizures that progressed from stage 1 (frozen and leaning) to stage 6 (fore- and hind-limb clonus) within 60 min after injection. During the remaining period of analysis, seizure scores stabilized around levels 5 and 6.

Compared to KA-injected WT mice, a significant improvement in behavioral outcome was obtained when Panx1 channels were blocked or deleted (Figs. 5A–F). The overall seizure score, measured as the mean±s.e.m. values of the areas under the curves (seizure score×time), was significantly decreased from 619.2±32.9 to 446.0±51.9 score×sec when Panx1 was blocked by i.p. injection of MFQ prior to KA injection (Figs. 5A, C). A significant decrease in the overall seizure scores, compared to that of WT mice, was also obtained for the two Panx1-null (Panx1^−/−^ and Panx1-KOfirst) mice; the mean±s.e.m. values of the areas under the curves for these transgenic mice were 328.3±62.0 and 319.3±72.5 score×sec (Fig. 5C). No significant difference in terms of overall scores was observed between the two Panx1-null (Panx1^−/−^ and Panx1-KOfirst) mice and between these transgenics and the MFQ-treated WT mice (Fig. 5C). The deletion or blockade of Panx1 channels did not alter the onset time to forelimb stiff (score 3; Fig. 5D) and marginally improved the worst seizure score (Fig. 5E). However, 60–70 min after KA injection, a substantial and progressive improvement of seizure scores were recorded, such that 2 hr after SE onset, seizure score significantly decreased from a median value of 6 (interquartile range: 4–6) to median values of 3 (interquartile range: 0–4) when Panx1 channels were blocked with MFQ, and to a median values 0.5 when Panx1 was deleted (interquartile ranges: 0–4 for Panx1^−/−^ and 0–1 for Panx1 KOfirst) (Fig. 5F). No significant differences were obtained between MFQ-treated and the two Panx1-null mice in terms of final seizure scores. These behavioral data indicate that Panx1 channels do not participate in the onset of KA-induced seizures, but substantially contribute to sustain and prolong seizure activity.

## Discussion

Using a variety of approaches and two transgenic mouse lines where Panx1 is deleted, we provide evidence that channels formed by this protein contribute to status epileticus *in vivo*. The data are consistent with a model in which the intense neuronal activity that occurs during epileptiform activity elevates extracellular K^+^. The increased extracellular concentration of this cation activates Panx1 channels, leading to the release of the excitatory transmitter ATP. The activation of purinergic P2 receptors resulting from the elevated extracellular ATP may further contributes to the neuronal hyperactivity, thereby initiating a positive feedback mechanism that accelerates the progression of seizures, worsens their degree and extends their duration.

Our results show that Panx1 channels are activated in hippocampal slices, as measured by the influx of the dye YoPro1, under conditions of neuronal hyperactivity. These findings are consistent with a previous report indicating opening of Panx1 channels in hippocampal neurons exposed to 100 µM NMDA potentiates seizure-like activity [5]. In that report, blockade of Panx1 channels with carbenoxolone or with the ^10^Panx1 peptide, as well as the knockdown of Panx1 expression with shRNA, was shown to prevent the NMDA receptor secondary current and dye uptake that occurred several minutes after the initiation of epileptiform discharges. The present study significantly extends these *in vitro* observations by showing that two independent lines of transgenic mice null for Panx1 did not display kainic acid-induced Panx1 channel activity. These novel *in vivo* data, together with the improvement of behavioral manifestations of seizure in the two Panx1-null mouse lines and in wild-type mice treated with MFQ, concur to indicate that Panx1 channels potentiate seizure activity.

The first evidence that Panx1 channels can be activated by elevated extracellular K^+^ concentrations was provided by Silverman et al. [10] using oocytes expressing Panx1 and a human astrocytoma cell line. Under voltage clamp conditions, currents sensitive to the Panx1 channel blocker carbenoxolone were shown to be significantly larger when oocytes were bathed in solution with elevated K^+^ [10]. In this same study, the high K^+^-induced influx of YoPro1 in 1321N1 astrocytoma was also shown to be totally prevented in cells in which Panx1 was knocked-down by shRNA.

Results obtained in the present study provide evidence that *in situ* and *in vivo* Panx1 channels can be opened by elevated K^+^ concentrations at levels similar to those reported to occur under intense neuronal activity [25]. Activation of Panx1 channels in hippocampal neurons and astrocytes that leads to dye uptake and ATP release from slices exposed to 10 mM K^+^ and from slices of KA-injected mice is here shown to be blocked by a potent agent and in two transgenic mouse lines lacking Panx1.

Although not fully established, ATP through its action on P2X receptors has long been proposed to be pro-convulsant [2], [26], [27], [28]. Increased levels of extracellular ATP have been recorded from hippocampal slices of rats susceptible to audiogenic seizures [2]. In this study, the authors detected, using the luciferin/luciferase assay, 0.79–1.52 pmol of extracellular ATP after stimulation of Schaffer collaterals of seizure-susceptible D2 mice, which was about 2 fold higher than that recorded from non-susceptible B6 mice. In agreement with this study, we also found comparable elevated ATP levels (0.31–0.36 nM: 1.55–1.80 pmol) in ACSF bathing brain slices exposed to high K^+^ and from those of KA-injected WT mice. Given that the amount of ATP present in the ACSF bathing the slices of Panx1-null mice was reduced to about 1.0 pmol, it is most likely that a substantial amount (30–40%) of released ATP occurs via Panx1 channels. Moreover, that Panx1-mediated ATP release is an important event that contributes to prolong hyperexcitabilty is provided from our results showing that suppression of Panx1 channels ameliorates the behavioral manifestations of status epilepticus.

In contrast to the excitatory action of ATP, adenosine has a well established anti-convulsant action by acting on adenosine A_1_ receptors. Activation of A_1_ receptor suppresses glutamate release from pre-synaptic terminals [29] and hyperpolarizes post synaptic neurons via ATP-sensitive potassium channels [30]. During seizures, extracellular adenosine levels rise substantially [31], due to the hydrolysis of ATP by ectonucleotidases [32], and to the release of adenosine through the equilibrative nucleoside transporters (ENTs) as a consequence of down-regulation/inhibiton of adenosine kinase [33], [34]. However, under conditions mimicking status epilepticus, it was shown that the loss of adenosine-mediated inhibition was correlated with the depletion of cellular ATP that occurred during the spontaneous switch between seizure-like events to late recurrent discharges [35]. It was also reported that such a cellular depletion of ATP results in the reduction of the phosphorylation state and function of the GABA-A receptors leading to increased number of after-discharges [36]. Thus, based on these reports, we can speculate that the beneficial effect seen here in the Panx1-null mice could be resultant from the prevention of total loss of cellular ATP that impairs animal recovery from prolonged seizures. Alternatively or in addition, it is also possible that the reduced release of ATP from Panx1-null mice could also limit the activation of excitatory P2X receptors and thus the progression of status epilepticus.

In summary, our results provide the first direct evidence that Panx1 channels worsen seizures and sustain status epilepticus *in vivo*. While further studies are now required to differentiate the respective contributions of the neuronal and glial Panx1 and to conclusively determine the contribution of ATP/P2X receptors to this deleterious condition, the data open new perspectives for the development of innovative therapeutic approaches which, by targeting the Panx1, may be beneficial for the prevention and/or treatment of status epilepticus.

## Supporting Information

Figure S1 **Characterization of two different Panx1-null mice (Panx1^−/−^ and Panx1 KOfirst).** (A) PCR of mouse tail DNA using primers specific for either the wild type or the mutated allele, distinguished wild type (+/+), heterozygous (+/−) and homozygous null Panx-1 mice (−/−). (B) Quantitative RT-PCR showing the expression levels of Panx1 in the hippocampus of Panx1^+/+^ (black bar), Panx1^+/−^ (grey bar) and Panx1^−/−^ mice (open bar). Values are mean ± s.e.m. levels of the number of mice indicated. *** p<0.001 Panx1^+/−^ *vs* Panx1^+/+^ mice, Panx1^−/−^ *vs* Panx1^+/−^, and Panx1^−/−^ *vs* Panx1^+/+^ mice. Three animals of each genotype were used. (C) Tail PCR products obtained using specific primers designed to detect the wild type (WT) 579 bp, the homozygous Panx1 KO-first (KO) 381 bp amplicons and the heterozygous (HT) 579 and 381 bp amplicons. (D) Western blot performed on cell lysates of cultured astrocytes obtained from two WT and three Panx1 KOfirst neonate mice using anti-Panx1 antibodies. Membrane was re-probed with anti-GAPDH.
(TIF)
