# Supplementary figures and images for: Targeting Pannexin1 Improves Seizure Outcome
Source: PLoS One. 2011 Sep 16;6(9):e25178. doi: 10.1371/journal.pone.0025178 (PMC3175002; doi:10.1371/journal.pone.0025178)

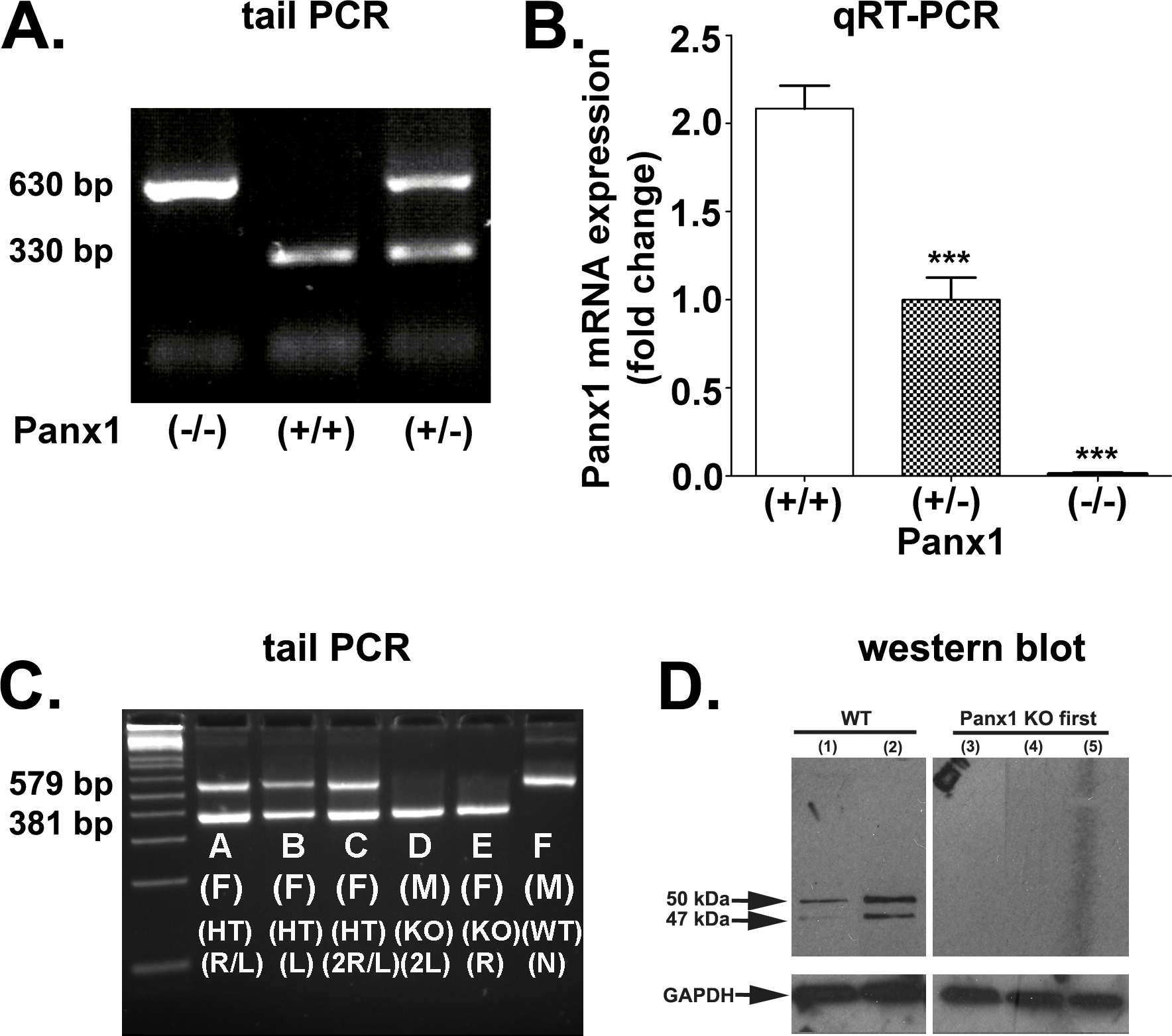

Supplement: Figure S1 — Characterization of two different Panx1-null mice (Panx1−/− and Panx1 KOfirst). (A) PCR of mouse tail DNA using primers specific for either the wild type or the mutated allele, distinguished wild type (+/+), heterozygous (+/−) and homozygous null Panx-1 mice (−/−). (B) Quantitative RT-PCR showing the expression levels of Panx1 in the hippocampus of Panx1+/+ (black bar), Panx1+/− (grey bar) and Panx1−/− mice (open bar). Values are mean ± s.e.m. levels of the number of mice indicated. *** p<0.001 Panx1+/− vs Panx1+/+ mice, Panx1−/− vs Panx1+/−, and Panx1−/− vs Panx1+/+ mice. Three animals of each genotype were used. (C) Tail PCR products obtained using specific primers designed to detect the wild type (WT) 579 bp, the homozygous Panx1 KO-first (KO) 381 bp amplicons and the heterozygous (HT) 579 and 381 bp amplicons. (D) Western blot performed on cell lysates of cultured astrocytes obtained from two WT and three Panx1 KOfirst neonate mice using anti-Panx1 antibodies. Membrane was re-probed with anti-GAPDH. (TIF) [file pone.0025178.s001.tif]
